# Skin-Associated B Cells in the Pathogenesis of Cutaneous Autoimmune Diseases—Implications for Therapeutic Approaches

**DOI:** 10.3390/cells9122627

**Published:** 2020-12-07

**Authors:** Tanja Fetter, Dennis Niebel, Christine Braegelmann, Joerg Wenzel

**Affiliations:** Department of Dermatology and Allergy, University Hospital Bonn, 53127 Bonn, Germany; tanja.fetter@ukbonn.de (T.F.); dennis.niebel@ukbonn.de (D.N.); christine.braegelmann@ukbonn.de (C.B.)

**Keywords:** autoimmunity, skin, B cells, autoantibodies, tertiary lymphoid structures, BLyS, BAFF, lupus erythematosus, pemphigus, pemphigoid

## Abstract

B lymphocytes are crucial mediators of systemic immune responses and are known to be substantial in the pathogenesis of autoimmune diseases with cutaneous manifestations. Amongst them are lupus erythematosus, dermatomyositis, systemic sclerosis and psoriasis, and particularly those driven by autoantibodies such as pemphigus and pemphigoid. However, the concept of autoreactive skin-associated B cells, which may reside in the skin and locally contribute to chronic inflammation, is gradually evolving. These cells are believed to differ from B cells of primary and secondary lymphoid organs and may provide additional features besides autoantibody production, including cytokine expression and crosstalk to autoreactive T cells in an antigen-presenting manner. In chronically inflamed skin, B cells may appear in tertiary lymphoid structures. Those abnormal lymph node-like structures comprise a network of immune and stromal cells possibly enriched by vascular structures and thus constitute an ideal niche for local autoimmune responses. In this review, we describe current considerations of different B cell subsets and their assumed role in skin autoimmunity. Moreover, we discuss traditional and B cell-associated approaches for the treatment of autoimmune skin diseases, including drugs targeting B cells (e.g., CD19- and CD20-antibodies), plasma cells (e.g., proteasome inhibitors, CXCR4 antagonists), activated pathways (such as BTK- and PI3K-inhibitors) and associated activator molecules (BLyS, APRIL).

## 1. Introduction

B cells represent an indispensable component of humoral immunity as they have the capacity to produce antibodies, which contribute to systemic inflammatory responses. Following traditional considerations of B cell functions in cutaneous autoimmunity, autoreactive B cells may possibly be generated in the bone marrow (BM) or secondary lymphoid organs (SLO) [1,2]. For instance, BM-derived naïve B cells, which respond to self-antigens despite central tolerance checkpoints, are released into the blood, circulate to secondary lymphoid organs such as the spleen or lymph nodes and may also escape peripheral checkpoints [3]. Moreover, pathogenic autoantibodies can be generated in the germinal center of SLO [4,5], entailing clonal expansion of autoreactive B cells. These cells can differentiate into memory B cells, which provide a rapid response to subsequent autoantigen exposure, and plasma cells (PCs), which contribute to disease systemically by secretion of autoantibodies. PCs have the ability to return to the BM, where they can colonize and produce autoantibodies for a long period of time, maybe even for decades [6]. In several cutaneous autoimmune diseases (CAD), the presence of autoantibodies is considered a diagnostic criterion and moreover, in some of these disorders a pathogenic role for specific autoantibodies is well described [7,8].

In addition to this, there is growing evidence for B cells distinct from B cells of primary or secondary lymphoid organs, which reside and act in the skin [9]. The skin marks the interface between the body and the environment and is in constant interaction with potentially harmful structures and pathogens. Various players of the innate and adaptive immune system constantly “patrol” the epidermis and dermis to induce inflammatory reactions if necessary [10]. Antigen-presenting cells (APC) including epidermal Langerhans cells play a key role in this regard and skin homing of T cells represents another line of defense. Apart from this, even in healthy skin, some neutrophils, monocytes and, as already mentioned, interspersed B cells are detectable as well [11]. This finding is quite surprising as B cells normally “belong” to primary or secondary lymphoid organs, which the skin is not by definition. Yet, in certain inflammatory conditions, specific leukocyte clusters may arise in the skin. These B cell populations migrate to the skin to locally produce cytokines and possibly autoantibodies which may contribute to disease amplification or amelioration depending on the respective microenvironment. Further presumed features include antigen presentation and participation in tertiary lymphoid structures (TLS) at the site of inflammation [9,12].

Differences in the distribution, number and function of B cell subtypes might be partly accountable for disease severity and altered response to therapy. Although underlying mechanisms of CAD have been widely studied in different murine and human models, the exact role and effects of autoreactive B cell subsets in the skin remains elusive.

In this review, we will dissect the current pathological concepts of Bcell autoimmunity in several inflammatory skin diseases focusing on cutaneous lupus erythematosus (CLE) and autoimmune bullous dermatoses (pemphigus and pemphigoid). We aim to establish an apprehensive overview of B cell subsets involved in CAD, the development of autoreactive B cells as a general concept and their potential features in skin autoimmunity. After all, a better understanding of the underlying mechanisms might pave the way for individualized and targeted therapies for different CAD, an aspect which will be covered in the final section of the manuscript. The information given is based on selective literature search of pubmed (search terms: cutaneous autoimmune diseases, skin autoimmunity, B cells (and subsets), tertiary lymphoid structures), Clinicaltrials.gov (search terms: pemphigus, pemphigoid, cutaneous lupus erythematosus, dermatomyositis, systemic sclerosis and chronic graft versus host disease) and our own experiences.

## 2. Introduction to Autoreactive B Cell Development and B Cell Subsets Described in CAD

### 2.1. Development of Autoreactive B Cells: Background Information on General Mechanisms

The majority of B cells originate from common multipotent hematopoietic stem cells (HSCs) within specialized niches in the BM. In these niches, HSCs mature into common lymphoid progenitors, which develop into pre-pro-B cells, pro-B cells, pre-B cells and lastly immature IgM+ B cells [13,14]. Central tolerance mechanisms include clonal deletion, receptor editing and anergy to ensure the elimination of autoreactive B cells in the BM. However, not all of these cells are filtered out before they leave the BM [15,16]. In SLOs, B cells can respond to an antigen in a T-dependent and T-independent manner, supported by an appropriate milieu of cytokines such as B lymphocyte stimulator (BLyS), also named B-cell activating factor (BAFF), and A proliferation-inducing ligand (APRIL) [17,18]. B cells can present the internalized and processed antigen via class II major histocompatibility complex (MHC) molecules, thus enabling the interaction of B cells with antigen-specific, priorly activated T helper (Th) cells in the paracortex of the lymph node. With the help of Th cells, B cells mature into short-lived immunoglobulin (Ig)M-producing plasmablasts, residing in the medullary cord, or B cells, participating in the formation of a germinal center (GC). Here, they undergo somatic hypermutation (SHM), affinity maturation and isotype switching with the assistance of follicular dendritic cells and T follicular helper (Tfh), the latter by receptor–ligand interactions (ICOSL–ICOS; CD40L–CD40) and cytokines, particularly IL-21 [19,20,21,22,23,24,25]. During GC maturation, the loss of self-tolerance can occur by inappropriate filtering of autoreactive cells or newly aquired autoreactivity [26,27]. As a result, memory B cells and PCs are generated, which contribute to the inflammatory process in CAD and will be discussed further on [28].

### 2.2. Overview of B Cell Subsets Described in CAD and Their Potential Role in Skin Autoimmunity

B cells can be classified into several subtypes, including innate-like B cells (ILB) such as B-1 cells, conventional B-2 cells, memory B cells, PCs and regulatory B (Breg) cells [29,30,31,32,33]. Although the understanding of the different nature of B cells is rapidly evolving, the number of B cell subtypes and the specific role in skin autoimmunity have to be further investigated. First, we will describe each B cell subset in the broader context of autoimmunity and inflammation and then reflect on current ideas of potential functions in the skin.

#### 2.2.1. B-2 Cells

Conventional B (B-2) cells, also named follicular B cells, represent the most common subtype of B cells, which are known for their critical contribution to adaptive immune responses as they give rise to PCs and memory B cells [34].

For several autoimmune diseases involving cutaneous manifestations, such as pemphigus vulgaris (PV), SLE, systemic sclerosis (SSc) and psoriasis, B-2 cells have been described in the skin [35,36,37,38,39,40]. In some disorders such as pemphigus and SSc, disease severity and progression positively correlate with the number of infiltrating B cells [37,38]. Apart from that, B-2 cells have been found in inflammation associated with skin malignancy (squamous cell carcinoma, primary melanoma, melanoma metastasis) which might be a predictive marker for therapy response [41].

#### 2.2.2. Memory B Cells

Memory B cells are generated during T-dependent and T-independent responses to the respective antigen [42] and are able to persist for a long time span awaiting antigen reencounter [43,44].

In CAD, stimulated memory B cells contribute to inflammation by cytokine expression and antigen presentation to T cells besides the production of autoantibodies [45,46]. Interestingly, memory B cells can survive independently of anti-apoptotic factors including BLyS and APRIL [47], thus they have been shown to be resistant to therapeutic strategies targeting these factors [48]. In pemphigus patients, desmoglein (Dsg)3-specific memory B cells were found to be significantly increased during remission after immunosuppressive therapy and thus probably contribute to subsequent disease relapse [49,50]. This led to the hypothesis, that the Tfh cell tolerance checkpoint within the GC response might be altered entailing generation of autoreactive memory B cells in pemphigus patients [51]. The role of memory B cells in the skin, however, still remains elusive.

#### 2.2.3. Plasma Cells (PCs)

PCs can arise due to the activation of naïve B cells, either rapidly generated in extrafollicular locations such as the medullary cord or in the GC [52]. PCs may generate persistent autoantibody titers in the absence of the antigen as long as they receive survival signals such as BLyS, APRIL and IL-6 from adjacent cells in their specific niches in the BM, SLO and at sites of chronic inflammation [53,54,55,56,57,58,59,60,61].

In inflammatory and autoimmune skin diseases, associated antibodies have generally been believed to be of systemic origin, implying their generation by PCs residing in either the BM or SLO such as lymph nodes. Apart from that, a few studies indicate a localized secretion of antibodies at the site of inflammation and even in healthy human skin, in which PCs secreting IgA antibodies were found to reside at eccrine sweat glands [62,63,64]. Skin-residing PCs may develop independently of T cells and accumulate in inflamed skin [65,66]. They are dependent on survival and proliferation-stimulating factors such as BLyS and APRIL, which have been shown to be upregulated in inflammatory skin [67,68]. Moreover, they are able to reside at sites of chronic inflammation for up to a lifetime. In SSc patients, a significantly enhanced number of CD138+ PCs was detected in lesional skin [38]. In pemphigus patients, CD19+ B cells and CD138+ PCs were found to be significantly increased in lesional skin and these isolated lymphocytes produced pathogenic anti-Dsg1 and anti-Dsg3 antibodies in vitro [37]. Accordingly, it has been suggested, that not only systemic but also localized (auto)antibody secretion by skin-resident PCs might contribute to chronic inflammation [9].

#### 2.2.4. Innate like B Cells (ILBs)

ILBs comprise a heterogeneous group of B cells, which are commonly known for their immunomodulatory properties during early innate immune responses. These presumed features include expression of proteins such as IL-10, IL-3, IL-35, adenosine and granulocyte-macrophage colony-stimulating factor (GM-CSF), antigen presentation to T cells and most importantly spontaneous secretion of natural low-affinity antibodies, predominantly IgM antibodies by the ILB subgroup named B-1 cells [69], in a T-independent manner [69,70]. In mice, B-1 cells are postulated to predominantly reside in the peritoneal and pleural cavities and at mucosal sites [71]. B-1 cells can be found in human healthy skin and in increased numbers in inflamed skin. They provide a constant level of immunoglobulins and thus participate in the initial defense against pathogens [62,72]. Since their natural antibodies can be self-reactive, B-1 cells are also believed to ensure rapid phagocytosis of endogenous danger associated molecular patterns, such as cell debris, to prevent the stimulation of pattern recognition receptors and thus initiation of autoimmune responses [73]. Accordingly, in a lupus prone mouse model, deficiency of natural IgM secretion accelerated the production of IgG autoantibodies and increased disease severity and progression [74]. Paradoxically, in SLE patients, numbers of B-1 cells were elevated and expressed high levels of costimulatory molecules CD80/86, suggesting proinflammatory activity of B-1 cells via increased T cell stimulation in this context [75,76,77]. According to these findings, dysregulated homeostasis of ILBs probably contributes to the initiation of autoimmune diseases, although their role in cutaneous autoimmunity has to be further determined.

#### 2.2.5. Regulatory B Cells (Bregs)

Bregs constitute a heterogeneous subgroup. They are known to express immunoregulatory cytokines such as IL-10, IL-35 and TGF-β and thereby induce regulatory Foxp3+ T cells [78,79,80,81,82]. Thus, they represent relevant players in the balance of skin immunity. In mice and humans, several subtypes are described reaching from innate Bregs to regulatory PCs, suggesting that Bregs are not a separate cell lineage and regulatory functions can be acquired at several stages of differentiation [83,84,85,86]. The basic capability of B cells to sufficiently suppress skin inflammation has been described first for hypersensitivity skin reactions nearly fifty years ago [87,88]. Recently, Bregs were also found to reside in healthy human skin and continuously express IL-10 even in the absence of inflammation. These Bregs represented innate-like (B-1) cells, which were shown to migrate to the skin via interaction with α4β1-integrins [89].

In several CAD, the specific state and function of Bregs appear to vary: in Psoriasis, deficiency of peripheral blood Bregs leads to significantly decreased expression of IL-10, enabling an upregulated expression of proinflammatory cytokines, amongst them are IFN-y and IL-17 produced by Th1- and Th17-cells, respectively. Accordingly, therapeutic drugs such as Rituximab (RTX), which lead to an unselective depletion of all B cell subsets including protective Bregs, have been found to induce or aggravate psoriatic skin lesions [90,91,92,93,94,95]. In patients with dermatomyositis, remarkably decreased numbers of peripheral blood Bregs compared to healthy controls were observed, which correlated with disease severity [96]. In contrast, in PV patients, Bregs were found to be increased in the peripheral blood but exhibited defective suppressive functions on Th1 cells [97]. Thus, the underlying potential triggers and regulatory mechanisms leading to these discrepancies still need to be clarified.

## 3. Potential Features and Clustering of B Cells in Lesional Skin

### 3.1. Role of B Cells as APC in Autoimmunity and Their Potential Contribution to Skin-Driven Inflammatory Responses

B cells can also function as APC as they express MHC II, costimulatory molecules including CD40 and CD80/86 and cytokines such as IFNγ, which are required for the activation of CD4+ T cells [98,99,100,101,102,103]. Stimulated T cells, in turn, further drive the inflammatory process via secretion of potent cytokines like IFN-γ, TNF-α, IL-4, IL-13 or IL-17 among others depending on the specific subtype [104,105]. In SLE, B cells are believed to additionally function as APC that can enable autoreactive T cell activation due to observations in a lupus prone mouse model: mice reconstituted with B cells lacking the capacity to produce antibodies still developed lupus-like disease in contrast to mice completely depleted of B cells, in which no disease occurred [106]. Consistently, lupus prone mice with a B cell-specific MHC II deletion also showed markedly ameliorated disease [107]. In addition, therapeutic strategies targeting B cells or their activator molecules such as BLyS have been shown to alter T cell functions as observed in pemphigus patients: treatment with the anti-CD20-antibody RTX resulted in a significant decrease in Dsg-specific, IL-4- and IFNγ-producing autoreactive T cells in the peripheral blood which is ascribed to the deletion of B cells that enable T-cell activation as critical APC [108]. Targeting B cells can also be successful in diseases that are primarily considered T cell-mediated or frequently lack autoantibodies, such as chronic discoid lupus erythematosus [109,110], supporting the idea of an additional function of B cells in CAD, in addition to autoantibody production. Skin associated B cells, either recirculating or resident, were also found to express high levels of MHC II and costimulatory molecules (CD80/86) [62], which raises the hypothesis of a specific antigen-presenting role and interaction with co-localizing T cells in the skin. However, it needs to be further determined which phenotype of B cells is responsible for this feature. B cells have been shown to cluster in the dermis or participate in the formation of TLS [37,111,112]. These structures, consisting of other immune cells such as T cells, follicular dendritic cells and macrophages, stromal cells as well as several cytokines provided by the respective cells, might have the potential to create an appropriate micromilieu for skin-localized autoantigen-driven immune responses, as discussed below.

### 3.2. B Cells in the Formation of TLS

#### 3.2.1. Historic Origins

Formation of lymphatic aggregates in the skin were first described in 1978 by Streilein et al. who named it “skin associated lymphatic tissue” (SALT) and further characterized it in the following years [113,114]. As formation of lymphatic tissue in the skin requires specific, context dependent inflammatory stimuli, it is now most commonly referred to as “inducible skin associated lymphatic tissue” (iSALT) [115]. In the skin, B cells may cluster and aggregate in direct interaction with T cells to form lymphoid structures. Of note, similar reactions have been described in other organs (e.g., liver, kidney, heart, salivary glands) in the course of infection, severe autoinflammation or malignancy. In an attempt to generally explain the findings of lymphoid structures in extralymphatic organs, the terms “tertiary lymphoid structures” (TLS) or “tertiary lymphoid organs” (TLO) were introduced. Although often named TLO, these structures may not fulfill the appropriate definition of an organ as they lack a structural organization and capsule [116]. The existence of TLS in the skin and a clear discrimination to iSALT is still topic of debate as some authors consider iSALT to be TLS. ISALT most commonly appears in response to hapten application on the skin which then leads to interaction between dermal dendritic cells and T cells followed by recruitment of effector T cells; they generally lack B cells. TLS may also be understood as proper lymphatic tissue which will be described in the following sections.

#### 3.2.2. Physiology of TLS

TLS, by definition, consist of separated zones of B cells and T cells which form clusters with follicular dendritic cells pivotal for providing survival factors. Other features such as high endothelial venules indispensable for T-cell trafficking and lymphoid stromal cells can be detected as well [117,118,119]. Germinal centers may be present [111]. In contrast to SLO, TLS lack an organized lymphatic system, yet the presence of lymphatic vessels has been described [117]. In terms of the induction of humoral responses, normally, interaction of naïve B cells with specific T cells leads to an antigen switch and formation of PCs and, subsequently, the production of antibodies. However, given the proximity to the site of inflammation, it has been proposed that in TLS, antigen presentation might occur via diffusion rather than dendritic cells [120]. The delicate architecture of primary and secondary lymphoid tissues is not well established in TLS and generally, TLS may be considered transient structures as they often disappear subsequent to antigen clearance. It is noteworthy that TLS are absent in embryonic life and only form depending on internal and external stimuli.

#### 3.2.3. TLS Formation and Associated Inflammatory Cytokines

Internal factors associated with TLS formation as responses to chronic lesional inflammation in general include a cytokine signature of lymphotoxin and TNF in combination with lymphokines CCL19, CCL21 and CXCL13 [37,121,122]. The process depends on the interplay of different mesenchymal cells, e.g., stromal cells, and infiltrating immune cells: via lymphotoxin-β receptor signaling, lymphoid cells can induce expression of adhesion molecules and respective lymphokines in mesenchymal cells. These mediators, in turn, initiate and regulate the formation of TLS [121,122]. TLS formation independent of lymphotoxin, however, has also been described [123]. Once TLS are established, a milieu rich in survival factors, e.g., BLyS, IL-6, IL-7 and CXCL12 enables the persistence of incoming PCs and thus makes them inaccessible for systemic approaches of B cell depletion [124,125]. This finding is supported by a recent investigation of ectopic lymphoid-like structures in inflamed pemphigus lesions [112] and by the fact that isolated lymphocytes of lesional skin (as mentioned earlier) held the capacity to produce anti-desmoglein antibodies in vitro [37]. Of note, IL-22 and IL-17—mainly expressed by T cells—are also believed to play an important role in the formation or maintenance of TLS as they were shown to increase the expression of chemokines such as CXCL12 and CXCL13 in epithelial and/or stromal cells [126,127].

#### 3.2.4. TLS Formation Provides a Niche for Local Autoimmunity

**Figure 1:**
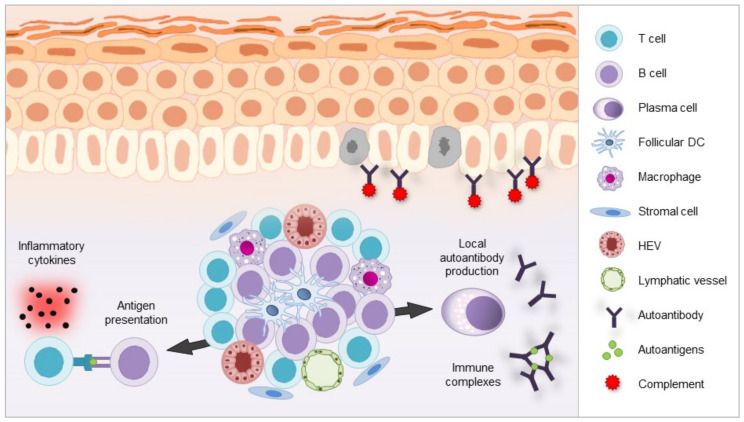
Schematic illustration of a potential tertiary lymphoid structure (TLS) in skin autoimmunity. TLS represent accumulations of lymphoid and stromal cells arising at ectopic sites as a reflection of chronic inflammation. As depicted, they may consist of B- and T-cell clusters and a network of stromal cells, follicular dendritic cells (DC) and macrophages as well as vascular structures such as high endothelial venules (HEV) and lymphatic vessels, possibly forming GC-like structures [12,116,117,119]. TLS may create a microenvironment that enables localized autoantigen-directed immune responses such as T cell activation, which might also be driven by B cells with antigen-presenting features. Proinflammatory cytokines are subsequently released from activated T cells and autoantibody production by PCs as well as immune complex formation are iniated [124]. TLS formation has been described in pemphigus and lupus erythematosus panniculitis, yet data concerning the appearance and specific role of TLS in autoimmune skin diseases are limited [111,112,133].

Although most antibodies are produced in primary or secondary lymphoid organs such as the BM and lymph nodes, the skin has the potential to act as a niche for localized antibody production. Moreover, it is known that skin-produced autoantibodies are crucial players in the pathogenesis of several CAD such as pemphigus and bullous pemphigoid (BP) [37]. As the typical architecture of primary and secondary lymphoid tissues has not been established, it has been hypothesized, that key checkpoints for autoreactive cell screening are not sufficient in TLS while an environment rich in survival factors, as described above, is provided [128,129]. This has led to the concept of “local autoimmunity”, which can be difficult to breach therapeutically as the persistence of TLS despite treatment with B cell depleting drugs has been reported in several inflammatory conditions [130,131]. This is why TLS might be relevant not only in later stages of disease, but also in early phases when self-reactivity must be excluded via fully-functional control mechanisms. In the course of disease, TLS may act as a site of restimulation of memory lymphocytes or priming of precursors that boost differentiation and expansion of effector cells and maintain self-reactive inflammatory responses [112,124]. B cells can act as important APC in this regard and promote the expansion of Th cells [132]. The possible components of TLS, which may arise in the skin in response to chronic inflammation in CAD, are shown in Figure 1. However, these etiological concepts remain controversial until now, especially as data on CAD are very limited when compared to other autoimmune diseases, for instance rheumatoid arthritis or Sjögren’s syndrome [124].

## 4. Traditional and Targeted Therapeutic Strategies in CAD

CAD are numerous and heterogenous, yet the exact pathogenic role of B cells is under-investigated in most pathogenic disease models when compared to the role of T cells. Diseases which are defined by detectable autoantibodies in the serum have evoked more scientific attention in this regard. Two exemplary CAD are introduced below to outline current and future therapeutic modalities targeting B cells.

Autoimmune skin blistering diseases (AIBD) comprise a heterogeneous group of disorders defined by circulating autoantibodies against structural components of the epidermis or the dermo-epidermal junction. AIBD can be subdivided into pemphigus- and pemphigoid groups [134]. The most common subtype of the pemphigus group is PV, which is characterized by pathogenic anti-Dsg antibodies leading to acantholysis. Thus, PV clinically presents with flaccid blistering or erosions of oral mucosa (anti-Dsg 3) and the skin (anti-Dsg 1 and anti-Dsg3). In pemphigus foliaceus (PF), nearly all antibodies target Dsg1 leading to subcorneal blistering in the skin only. In regard to the pemphigoid group, BP is the most common subtype, in which antibodies against the hemidesmosomal proteins BP180 and BP230 can be found. This destruction of the dermoepidermal adhesion clinically results in tense blisters, which can also occur subsequent to a non-bullous phase of eczema-like, or urticarial skin lesions [134]. As several mentioned autoantibodies in pemphigus and pemphigoid are sufficient to cause loss of keratinocyte cohesion or dermoepidermal integrity, as shown in vivo, autoreactive B cells are well accepted as crucial players in the pathogenesis of AIBD [135,136,137]. Both pemphigus and pemphigoid group dermatoses generally have a major impact on the patients, as severe pain and pruritus significantly deteriorate the quality of life of the individual and as the course is chronic relapsing lacking proper therapy [138,139,140].

CLE is another CAD showing a broad spectrum of skin manifestations, which may occur as an isolated skin condition or as a symptom of systemic lupus erythematosus (SLE). CLE can be further subdivided into four subsets including acute, subacute, intermittent and chronic CLE (ACLE, SCLE, ICLE and CCLE, respectively). ACLE is characterized by a widespread maculopapular rash or facial indurated erythematous lesions and is often accompanied by systemic manifestations. SCLE may present with either papulosquamous or annular skin lesions, which preferentially occur in sun-exposed areas. In both ACLE and SCLE, autoantibodies (e.g., antinuclear antibodies or anti- double-stranded DNA antibodies in ACLE and anti- SSA/Ro or anti-SSB/La antibodies in SCLE) are a frequent phenomenon. ICLE presents with non-scarring and non-scaling erythematous plaques. CCLE can be classified into chronic discoid LE (CDLE), which presents with isolated plaques in a disc-like shape and may lack autoantibodies, LE panniculitis (LEP), involving lesions of the subcutaneous fat tissue, and chilblain LE, a rare variant presenting with erythematous papules or plaques located on acral areas [110]. On a molecular basis, the disease is characterized by a cytotoxic lesional immune response entailing the release of cellular debris, which is followed by re-activation of innate and adaptive immune pathways [141]. These include nucleic acid sensing, antigen presentation, leukocyte transendothelial migration and T and B-cell receptor signaling among others [142]. B cells are assumed to contribute to chronic inflammation in CLE skin lesions, although it remains elusive if—and which—B cell functions are of particular importance. They are believed to provide additional abilities besides autoantibody expression such as antigen presentation to autoreactive T cells [105] and cytokine expression [143]. Moreover, in several CLE subtypes, B cell activation- and survival-factor BLyS was shown to be strongly upregulated in lesional skin [67] and in LEP, B cells were found to participate in TLS [111,133], suggesting that they might also contribute to localized immune responses.

Traditional therapeutic approaches in CAD include the use of glucocorticoids and classic immunosuppressant drugs. The available data on the effects of these drugs on specific B cell subsets in different CAD are limited. Generally speaking, most traditional immunosuppressants deploy broad effects on multiple leukocyte subsets. This bears the chance to ameliorate symptoms of autoimmunity while increasing the risk of severe infections at the same time. We will first shortly introduce well established drugs for therapy of CAD to then discuss novel targeted treatment approaches.

### 4.1. Traditional Therapeutic Approaches in CAD

Corticosteroids have a multiplicity of immunomodulating, immunosuppressive and anti-inflammatory effects and traditionally represent a hallmark therapy of inflammatory dermatoses [144]. Altered transcription leads to transrepression of a wide range of proinflammatory cytokines, e.g., nuclear factor kappa-light-chain-enhancer of activated B cells (NF-κB), IFN-γ, IL-1, IL-2, IL-4 IL-6 and IL-13 [145]. Interestingly, the total number of circulating B cells decreases to a lesser extent than T cells and the production of antibodies themselves is only impaired by 10–20% upon corticosteroid therapy [146]. This is in line with the finding that corticosteroids have no major effect on long-lived PCs [129]. However, downregulation of IL-2 both reduces clonal B cell amplification and associated autoantibody production and cell-mediated immunity [147]. As therapeutic doses of glucocorticoids possess the capacity to quickly resolve B cell driven CAD [148,149], they have been and are widely used, despite the well described severe side effects in long term use including among others metabolic disruption, osteoporosis and increased risk of infections [144,150].

In addition to corticosteroids, several immunosuppressants can be used for the treatment of CAD. Antimetabolites including azathioprine, methotrexate and cyclophosphamide achieve a selective reduction in the number of lymphocytes which impairs both cellular and humoral immune responses. Limited data are available on the effects on different B cell subsets, however, it seems plausible that long-lived PCs are least affected, while the formation of new antibody secreting cells such as short-lived PCs is severely impaired [132,151]. Apart from their main mode of action, methotrexate in particular has a variety of immune-modulating off-target effects which have been reviewed elsewhere [152]. Azathioprine therapy decreases the number of cutaneous APC and T cells and hence interacts with B cell activation [147]. Cyclophosphamide has a relatively strong cytotoxic effect on lymphocytes and shows significant effects on lymphocyte derived cytokine expression [153]. Due to frequent severe side effects and both carcinogenic and teratogenic effects, it is only considered a salvage therapy in CAD [153]. Mycophenolate mofetil (MMF) allows a selective inhibition of inosinmonophosphat-dehydrogenase to reduce proliferation of lymphocytes, which are dependent on the de novo synthesis of purines [154]. MMF was also shown to suppress B cell proliferation and to reduce circulating PCs [155,156]. Side effects are generally less severe when compared to azathioprine or cyclophosphamide [157]. Originally developed to prevent transplant rejection in organ transplant recipients, it has been successfully used for over 20 years in refractory cases of CAD [158].

Another group of immunosuppressive drugs represent calcineurin inhibitors such as cylosporine, tacrolimus and pimecrolimus. These drugs are potent inhibitors of T cell proliferation, and thus indirectly impair B cell functions due to a reduced crosstalk with T-helper cells and T-follicular cells, which is necessary for autoantibody production. The availability of topical drug formulations is of specific interest in CAD, yet, the therapeutic effects of topical tacrolimus and pimecrolimus do not reach the efficacy of topical glucocorticoids [159].

### 4.2. B Cell Associated Therapeutic Strategies

There are several components associated with B cells that may function as therapeutic target: B cell surface molecules such as CD19 and CD20, B cell survival factors including BLyS and APRIL as well as signaling molecules, for instance Bruton’s tyrosine kinase (BTK) and phosphoinositide-3-kinase (PI3K) among others. Respective targeted treatment strategies come along with diverging features (Table 1), which are discussed in the following sections. Concerning inflammatory skin diseases, various drugs targeting these molecules are currently investigated in clinical trials (Table 2).

#### 4.2.1. Targeting B Cell Surface Molecules

Given the outstanding role of B lymphocytes in autoimmune diseases, tremendous effort has been undertaken to specifically and selectively target autoreactive cells. B lymphocyte antigens CD19 and CD20 are both inevitable structural components of the membranes of the majority of B cells [170]. They represent necessary components for B cell receptor (BCR) interactions and are suitable target structures both for diagnostic and therapeutic implications [171]. Given the fact that B cell malignancies such as B-NHL (B-Non-Hodgkin lymphomas) usually do not lose CD19 and CD20 expression, targeted therapies are well established for these indications [172,173]. CD19 is expressed on virtually the whole of the B cell compartment, including PCs while CD20 is expressed from pre-B cell to mature B cells. The most well studied and established drug is RTX as a first in-class monoclonal CD20 antibody [174]. Second generation CD20 antibodies have now been designed to improve their tolerability and effectiveness. Over the course of time this targeted approach has successfully been transferred from hematooncology to antibody driven autoimmune diseases including CAD (Table 1 and Table 2) [175,176]. Although CD20-directed therapies aimed to inhibit antibody production, their effect on B memory cells and long-lived PCs is slight. Notably, in some patients CD20 blockade yields worsening of disease, which points to the multiple roles of B cell subsets including Bregs in pathophysiology [132]. In line with the success of CD20 antibodies, CD19 antibodies are also under clinical investigation both for B-NHL and autoimmune diseases. Modern hemato-onocologic therapeutic approaches such as chimeric antigen receptor (CAR) T cell therapies often utilize CD19 as target antigen [177,178,179]. A similar approach labeled chimeric autoantibody receptor (CAAR) T cell therapy has been postulated as a therapeutic strategy in autoimmunity with the potential to address B memory cells and potentially PCs [180]. This approach is very attractive for CAD with a clearly defined autoantigen and corresponding disease severity such as pemphigus [147]. Considering the necessary tremendous logistic efforts and costs of these personalized treatment modalities, this approach has not entered clinical care in CAD. Bearing in mind the stunning achievements in the hemato-oncologic field, further development will be anticipated with great curiosity.

##### CD 20 Antibodies: RTX

RTX is a chimeric type I monoclonal antibody that induces broad B cell depletion via activation of caspases, complement-dependent cytotoxicity, antibody-dependent cytotoxicity and phagocytosis [147]. A major drawback of RTX is its immunogenicity that may result in type-I allergic reactions promptly after initiation of the infusion and the development of anti-drug antibodies that may neutralize the therapeutic effects over the course of therapy [181]. RTX was approved in Europe for B-NHL in the late 1990s and for rheumatoid arthritis in 2006. RTX has now achieved its first dermatological approval for therapy of pemphigus both in Europe and the USA based on the results of a recent study [182]. Although the effects vary between patients, an estimate of over 80% of patients may achieve complete remission [182]. While its use was first designated for cases refractory to standard therapy regimens based on corticosteroids and other immunosuppressants, it is now considered a first-line treatment in new onset pemphigus, as recommended in different guidelines [183]. Other clinical trials were designed to explore RTX use in similar indications such as BP and mucous membrane pemphigoid, one trial is actively recruiting for the rare condition of cicatricial ocular pemphigoid (Table 2). The beneficial capacity of RTX in CAD is most likely not only due to the reduction in autoantibody levels via a near complete depletion of circulating B cells. Presumably, overall reduction in most B cell subsets and decreased crosstalk with other immune cells unfolds a potent anti-inflammatory effect. Of note, the B cell compartment may not be completely depleted by CD20 antibodies, memory B cells especially seem to be very robust in this regard, as described earlier [132]. Bioavailability might be impaired in inflamed tissues resulting in a protected space for aberrant B cells [62].

Apart from monoclonal antibodies, drug conjugates represent another possibility to selectively attack CD20 expressing cells, a strategy which is already in use for certain B-NHLs (e.g., Ibritumomab-tiuxetan) [184]. If autoantibody producing B cell subsets were to be clearly distinguishable from healthy B cell populations by a certain target structure, specific drug conjugates might be designed to induce cell death without negative effects on healthy B cells.

##### Second/Third Generation CD20 Antibodies

In an attempt to overcome the immunogenicity of RTX, humanized and fully human monoclonal high affinity CD20 antibodies were designed, including Ocrelizumab and Ofatumumab. A myriad of clinical trials have explored safety and efficacy both in malignancy and autoimmunity [185]. However, very few clinical trials have been launched considering CAD. Two major phase III clinical trials of Ofatumumab use in pemphigus have been terminated early for financial reasons, yet successful use is documented in a case report unresponsive to RTX [186]. The high affinitiy anti-CD20 antibody Veltuzumab was also successful in a pemphigus patient who did not respond to RTX [147]. Higher affinity anti-CD20 antibodies might also help to target autoreactive B cells in immune-privileged niches. Yet, until now it remains unclear if the advantages of these newer drugs are sufficient to justify their use when compared to the now well-established RTX.

##### CD19 Antibodies

Exploration of monoclonal antibodies or drug conjugates targeting CD19 has seen major advances over recent years, especially considering B cell malignancies such as acute lymphoblastic leukemia [187,188]. CD19 expression is even more common across the B cell lineage: It is also detected on very early pro-B cells and PCs. As a crucial co-receptor of the B cell receptor, great attention has also been attached to CD19 in the context of antibody-dependent autoimmune disease [189]. The drug XmAb5871 has entered clinical trials for rheumatoid arthritis and multiple sclerosis and is a potential drug target in SLE (NCT02725515), hence it might become relevant for CAD, if its efficacy and tolerability are acceptable. The specific differences as well as advantages and disadvantages between targeting CD19 and CD20 still need to be defined. One concern regarding the depletion of long-lived PCs upon anti-CD19 antibody treatment, is that it might undesirably facilitate the abolishment of vaccinatione-derived neutralizing antibodies.

#### 4.2.2. Targeting B Cell Activation and Survival Factors

##### BLyS (BAFF)/APRIL Antagonists

In an approach to selectively deprive B cells without directly attacking them, inhibition of survival factors and their receptors has been implicated over the last decade [190]. A great deal of detailed bench work was necessary to define the complex role of BLyS and APRIL and their corresponding receptors and the fine-tuned effects on B cell maturation. The achieved successes lead to the approval of Belimumab in the EU and US for SLE refractory to standard therapy [191,192,193]. Treatment with this well tolerated antibody mostly affects naïve and activated B cells but antibody production is not fully impaired. Accordingly, the therapeutic potency of BLyS antibodies itself may not achieve remissions in SLE; however, it clearly has a beneficial steroid-sparing effect and appears to carry a less severe risk of infections when compared to CD20 antibodies [48]. Belimumab most probably bears the capacity to improve CLE, as BLyS was found to be significantly upregulated in lesional skin [67]. Belimumab is currently being further investigated in an actively recruiting multicenter clinical study initiated by our department (BELI-SKIN, EudraCT 2017-003051-35). We hereby aim to explore the safety and efficacy of the drug in subcutaneous application for CLE patients and strive to further define and specify the pathophysiological role of all involved effector cells including B cell subsets by extensive studies of inflammatory signatures in lesional skin and corresponding patient serum.

However, the therapeutic effects of BLyS inhibition may vary considerably between individuals and between diseases. Another BLyS inhibitor under investigation is Tabalumab, however, a major SLE study was terminated early as the endpoint was not reached (NCT02041091) and a large study regarding RA showed neither clinical efficacy nor relevant safety events [194]. APRIL is of specific interest as a target structure to deplete long-lived PCs which contribute most to antibody production when compared to other subsets of the B cell compartment [195].

Fusion proteins designed to neutralize BLyS (Blisibimod) and APRIL (Ataticept) have reached phase III studies in SLE. The results of a phase IIb trial of Ataticept are promising and more effective than BLyS inhibition alone [196], however, partly discouraging results for therapy of MS have hampered excitement [197]. Lanalumab is the only BLyS inhibitor currently under clinical investigation for another CAD, which is PV (NCT01930175). Preclinical data of a murine scleroderma model have shown positive effects of BLyS inhibition by modulation of the balance between regulatory and effector B cells [198]. So far, apart from PV, CAD have not evoked sufficient attention to spark initiation of clinical trials for any of these drugs, although based on our current understanding, beneficial effects appear conceivable.

#### 4.2.3. Targeting B Cell Signaling Molecules

##### BTK Inhibitors

Small molecules selectively targeting BTK have entered daily care of B-cell malignancies with Ibrutinib as a first in-class drug [199]. As a crucial player in the downstream cascade following B-cell receptor activation, BTKs exert actions towards B cell survival and maturation via p38MAPK, MEK/ERK and NFkB pathways [200,201]. A specific role of BTK has been postulated due to effects on B/T-cell crosstalk and loss of peripheral B cell tolerance [202]. PRN1008 is an orally available drug currently under investigation in a phase III trial for PV and other autoimmune diseases (NCT03762265). Another drug, Elsubritinib, is under investigation for treatment of SLE (NCT04451772) as preclinical data are promising [203]. Further exploration of possible therapeutic use will largely depend on the results in autoimmune diseases, BTK might evolve as an attractive therapeutic option in CAD if proven effective and safe.

##### PI3Kδ Inhibitors

PI3Ks are an essential protein family involved in numerous functions of cell cycle and growth [204]. The delta isoform constitutes a protein mainly expressed on hematopoietic cells including B cells [205]. It has a major role in the PI3K/AKT/mTOR pathway, which mediates signals from BCR [205], CD19 [206], chemokine receptors [207] and the BLyS receptor [208] in B cells and was shown to be crucial both in malignant neoplasms such as B-NHL [209] and autoimmunity [210] as it enables development, activation and survival of B cells [211,212]. PI3K inhibitors bear the potential to target innate-like B cells and B1 cells which are elemental in early stages of autoimmunity [205,213]. Promising preclinical data are available from murine RA and SLE models [214,215]. Concerning CAD, a study evaluating the use of PI3Kδ inhibitor Parsaclisib in PV was withdrawn due to low recruitment (NCT03780166). Promising preclinical data lead to a phase I study to investigate the safety and tolerability of Seletalisib in healthy volunteers and psoriasis vulgaris patients in 2014 [216,217]. More recent developments are expected in CAD.

##### SHIP1 Activators

Another candidate for therapeutic intervention is the Src homology 2 domain-containing inositol 5′phosphatase 1 (SHIP 1), which among other receptors affects the BCR by activation of the inhibitory FC gamma receptor IIB unfolding an immune-regulating impact [218,219]. SHIP1 is a multi-domain protein centrally involved in numerous inhibitory pathways and downstream effector cascades (including PI3K and BTK) and its exact function is incompletely understood despite extensive investigations [220,221]. Taken together, based on inhibitory actions on multiple activating pathways, it does play a role in dampening the activation and function of B/T-lymphocytes and other immune cells unfolding an immune regulating effect [222]. Preclinical data indicate that lack of SHIP1 may give rise to a lupus-like inflammation [223] and B cells from lupus patients showed decreased activity of SHIP1 when compared to healthy controls [224]. Therefore, activation of SHIP1 by targeted small molecules might be a therapeutic option in SLE and autoimmunity in general including CAD. A study evaluating the use of SHIP1 activator AQX-1125 (Rosiptor, NCT02324972) in mild to medium severe atopic dermatitis failed to demonstrate major efficacy after 12 weeks of treatment while displaying a satisfying safety profile. There are no further active clinical trials in CAD at this time; further preclinical data in models of skin autoimmunity are necessary first to clarify potential indications for therapeutic use.

##### ROCK2 Inhibitors

The Rho/Rho-associated coiled-coil containing protein kinase (Rho/ROCK) pathway is known to be involved in numerous cellular processes including B cell development, activation and survival [225]. For instance, the inhibition of ROCK was shown to partially blunt the response of normal B cells to BLyS [225]. It also plays a role in cytoskeletal re-organization and BCR-dependent proliferation of mature B cells [226]. The type two isoform of Rho-associated coiled-coil kinase (ROCK2) was shown to be crucial for the formation of GC B cell responses [227]. However, the exact role of ROCK2 in B cell biology remains to be clarified. Yet, there are actively recruiting clinical studies evaluating the use of the orally available ROCK2 inhibitor Belumosudil in diffuse cutaneous sclerosis (NCT03919799). Other indications of interest include graft-versus-host disease (GvHD) and psoriasis (NCT02852967); available data from phase II studies in psoriasis have shown promising results [228]. ROCK2 inhibitors might evolve as interesting agents in the management of different autoimmune diseases; however, a better understanding on the effects on cell subsets is crucial.

#### 4.2.4. Challenges of Therapies Targeting B Cells

There are several therapeutic strategies that target B cells and/or their activating ligands. This leads to a sufficient depression of matured B cells and plasmablasts, probably resulting in a slight decrease in autoantibody-titers. However, long-lived PCs, which most commonly reside in the BM in a specific survival-supporting niche, are not affected by these therapeutic strategies [151,229]. This results in persisting autoantibody-production and maintenance of the inflammatory process [230]. Selective targeting of long-lived autoreactive PCs remains a fundamental challenge as the concomitant depletion of protective PCs against pathogens such as pneumococci, tetanus and influenza would have severe implications [56,231].

An ideal approach to treat autoimmunity might be to specifically target autoreactive effector B cells while at the same time creating a relative outweigh of Breg effects. Therefore, even more detailed knowledge of the delicate interaction between different immune cell subsets is required, and tailored approaches for a specific condition and patient might require combinations of the aforementioned therapies.

#### 4.2.5. PC-Associated Therapeutic Strategies

It has been widely recognized that PCs represent a cell subset refractory to B cell depleting therapies [232]. Potential strategies targeting pathogenic PCs include, among others, the induction of apoptosis, the dislocation of PCs from immune-privileged niches and the disturbance of migration of newly formed PCs. Yet another point of action, is to eliminate pathogenic antibodies by apparatus procedures such as immunoadsorption. Again, some therapeutic approaches for autoimmune diseases may be transferable from the significant achievements in myeloma therapy.

##### Proteasome Inhibitors/Immunoproteasome Inhibitors

First in-class proteasome inhibitor Bortezomib bears the capacity to induce apoptosis in PCs via interaction of cell protein degradation. It has been used as monotherapy and combinatorial therapy in myeloma for more than a decade and is widely available [233,234]. It might represent a powerful tool to specifically eliminate long-lived PCs, which to some extent resemble myeloma cells concerning their metabolism [235]. Positive effects have been postulated in numerous autoimmune diseases such as experimental myasthenia gravis [236], SLE [237] and primary Sjögren’s syndrome [238], a case report describes the successful use in mucous membrane pemphigoid [239]. Another interesting approach is the attempt to selectively inhibit the so called “immunoproteasome”, the first in-class molecule KZR-616 is under clinical investigation for therapy of dermatomyositis (NCT04033926) and has already been used to treat SLE patients in early clinical trials [167]. It will be interesting to trail the development of these new compounds and its therapeutic use in rheumatic and cutaneous autoimmunity.

##### CXCR4 Antagonists

Chemokine interactions are essential for PC differentiation, survival and trafficking. The CXCL12–CXCR4 axis is a well-described key mechanism in this regard [240,241]. Binding of the ligand CXCL12 to its receptor CXCR4 on PCs orchestrates the traveling of plasmablasts from lymphoid organs to the bone marrow and represents a survival factor important for differentiation into long-lived PCs at the same time [242]. As outlined above, under inflammatory conditions, TLSs may act as immune-privileged niches for PCs with a micromilieu rich in survival factors. PC-directed therapies like proteasome inhibitors might be less effective in these privileged areas; therefore, there are therapeutic efforts to dislocate PCs from their niches. There are both peptide and non-peptide CXCR4 inhibitors available including AMD3100 (Plerixafor) and CTCE-9908 [241,243]. Both have shown promising results in preclinical data of murine SLE models. The prior has entered numerous preclinical and clinical trials for conditions ranging from hematological malignancies to diabetic ulcers [244,245,246]. Until now, monotherapy with CXCR4 antibodies appears to be a rather theoretical approach for the treatment of aberrant PCs; yet it is already an established therapy option for stem cell mobilization in certain B-NHL [247] and an evolving therapy option for numerous other cancers [248]. Combinatorial treatments might open another field of targeted therapy for antibody driven CAD.

##### FcRn Receptor Antibodies

The neonatal FC receptor consists of a H chain similar to the MHC class I and the β2-microglobulin L chain and is involved in the regulation of IgG degradation; FcRN expressing cells binding to IgG help to recycle the immunoglobulins rather than to degrade them which thereby leads to longevity of IgG [249,250]. In the presence of autoreactive IgG this process leads to persistence of autoimmune effects. Targeting the FcRn receptor is under clinical investigation in an attempt to enhance catabolism of aberrant immunoglobulins in autoimmune diseases, including pemphigus [147]. One phase II study was designed to investigate the effects of the neutralizing antibody efgartigimod (ARGX-113) in PV and PF (NCT03334058); the trial is active but currently not recruiting. Another drug in clinical development is monoclonal IgG4 antibody SYNT001 designed to disrupt the interaction of FcRn with IgG; a phase I/II study in PV and PF has been completed (NCT03075904), thus preliminary results are available only for the phase I part, so far.

Of note, donor derived intravenous immunoglobulin therapy (IVIG) which serves in a variance of severe cases in CAD via a multitude of immunomodulating strategies, also exerts effects via FcRn binding to eliminate aberrant and superfluous immunoglobulins [251]. It may show efficacy alone or in combination with other immunosuppressive strategies in CAD. The therapy relies on donations by healthy volunteers.

##### Plasmapheresis and Immunoabsorption

Plasmapheresis includes the elimination of immunoglobulins via plasma exchange ex vivo by selective filters. It therefore does not abrogate the production of new autoantibodies by PCs and is mainly helpful in conditions that directly depend on autoantibody titers such as pemphigus [252,253]. As other plasma proteins such as albumin and factors of hemostasis are eliminated, too, severe side effects including increased risk of sepsis, limit its use to severe and refractory cases of CAD. Further developments aim to lessen the negative effects of plasmapheresis by a relative selective depletion of immunoglobulins while sparing desired compounds of the plasma such as albumin (double filtration plasmapheresis (DFPP)). Promising results regarding efficacy and safety for this technique have repeatedly been reported in pemphigus patients from small case series [254,255]. Whether this procedure gains greater attention to spread into clinical practice will depend on various factors.

Comparably to plasmapheresis, immunoadsorption is an ex-vivo procedure to selectively reduce aberrant autoantibodies from the patient’s circulation. Specific filters with ligands binding IgG enable a more targeted filtration, yet immunosuppression is still considerable. Likewise, PCs in niches are not affected by therapy; however, life threatening conditions like pemphigus involving large areas of the body may quickly show improvement upon therapy [256,257].

## 5. Summary and Future Perspectives

A critical role of B cells is no longer only ascribed to known B cell-driven disorders with pathogenic autoantibodies, but their role is increasingly investigated in CAD lacking autoantibodies and even in diseases considered as being predominantly T-cell driven, as there is evidence for additional features of B cells, besides autoantibody production. They can induce autoreactive or regulatory T cells by the release of both pro- and anti-inflammatory cytokines depending on the subtype and microenvironment, thus either fueling or regulating the inflammatory process. B cells have also been shown to be well suited for antigen presentation to T cells due to their capability to express MHC II and costimulatory molecules. However, the specific phenotype and features of B cells associated with the skin in general and CAD still remain to be clarified, particularly to enable specific therapeutic approaches that take leading B cell subsets and associated molecular mechanisms into account. Furthermore, the established concept of systemic autoimmunity should be complemented by the idea of a localized inflammatory response of specialized TLS in the skin. These lymphoid niches may be considered in regard to therapeutic strategies as they can be difficult to target and thus, possibly enable continuous inflammation or a disease relapse. Therefore, a deeper understanding of TLS in skin autoimmunity, including: (i) the specific role of respective components and (ii) the factors, which lead to TLS development, maturation and potential GC formation; as well as (iii) regulatory mechanisms within these structures compared to SLO, is needed.

Due to the growing knowledge of B cells and their manifold ways to contribute to the perpetuation and regulation of inflammation in a broad spectrum of diseases (such as B cell malignancies and autoimmune diseases), numerous therapeutic strategies focusing on B cells, activated pathways and associated molecules have been developed and/or are currently under investigation. However, it remains an important future challenge to transfer these new therapeutic approaches, if proven safe and effective, to CAD. Several clinical trials investigating potential drugs for inflammatory skin diseases such as pemphigus and CLE are currently ongoing, which will hopefully provide insights regarding the specific therapeutic effect on different B cell subsets. A more precise characterization of involved cells and their exact role in skin autoimmunity will be pivotal for a personalized approach as a future perspective in the treatment of autoimmune skin diseases.

## Figures and Tables

**Table 1:** Exemplary B-cell- and plasma cell (PC)-associated therapeutic strategies and their advantages and disadvantages in regard to cutaneous autoimmune diseases. Another mode of therapeutically modifying B cell effects is via elimination of pathogenic immunoglobulins (e.g., plasmapheresis, immunoadsorption).

Target Cell	Target Structure	Mode of Action	Pro	Contra
Pro-B-cells–PCs	CD19 [160] e.g., XmAb 5871 (obexelimab)	Antibody-mediated depletion of a broad range of B cells including follicular dendritic cells and PCs to reduce autoantibody formation	Effects on autoreactive PCs and autoantibody production	Depletion of long-lived PCs and thus deprivation of protection provided by vaccination
Pre-B-cells–mature B cells	CD20 [161] e.g., MabThera (Rituximab)	Antibody-mediated depletion of peripheral B cells relatively sparing long-lived PCs Reduced production of inflammatory cytokines and activation of T cells	Potent therapeutic effect Biosimilars already available for first-in-class drug Rituximab Subcutaneous administration possible (Ofatumumab) “reset” of B cell compartiment may shift balance towards Bregs	Immunogenicity limits effect and safety in Rituximab Self-reactive B cells in immunologic niches (e.g., BM, TLS) may persist Severe immunosuppression; contraindication for live vaccination
Naïve and mature B cells	BLyS/ BAFF [162] e.g., Benlysta (Belimumab)	Antibody or fusion protein mediated reduction in B cell activation and survival of autoimmune B cells via reduced survival factors	Proposed relative selectivity for autoimmune B cells as they depend on overproduction of BLyS Might serve as a steroid sparing agent with good safety and tolerability	Medium potency effects; so far only add on therapy
B cells excluding PCs	BTK [163] e.g., Imbruvica (Ibrutinib)	Inhibition of BCR downstream signaling Initiation of apoptosis of aberrant B cells	Orally available Therapy well established in B cell malignancies; much experience More specific second generation inhibitors might be better tolerable	Common side effects limit its use, for Ibrutinib specifically increased risk of bleeding
B cells including innate like B cells and B1 cells, (T cells)	PI3Kδ [164] e.g., Zydelig (Idelalisib)	Selective inhibition of the isoform mainly expressed on hematopoietic cells which is crucial for B cell survival and proliferation	Orally available Promising pipeline of numerous compounds	Isoform specificity varies between different drugs Common side effects include skin reactions including severe cutaneous adverse events Paradoxical immune activation has been described as inhibition of T-reg cells is stronger than inhibition of T-eff cells
B/T cells, NK cells, mast cells, dendritic cells, macrophages	SHIP1 [165] e.g., AQX-1125 (Rosiptor)	Activation of SHIP1 leads to inhibitory interaction with BCR via different pathways, e.g., downregulation of PI3K signaling	Orally available Well tolerable	Negative clinical trials for other indications derogate expectations Multiplicity of cellular functions incompletely understood
B/T cells, other immune cells, non-hematopoietic cells	ROCK2 [166] e.g., KD025 (Belumosudil)	Inhibition leads to down/up regulation of central pro/anti-inflammatory interleukins (IL17/IL10) which leads to a decreased TH17 response and anti-inflammatory regulation of B cell subsets	Orally available Promising pipeline of numerous compounds Postulated beneficial effects on cardiovascular system	Involvement in numerous biological processes makes off-target side effects likely
PCs	Proteasome [132] e.g., Velcade (Bortezomib)	Enhanced apoptosis of PCs via disruption of intracellular protein degradation	Orally available Therapy well established in myeloma therapy; much experience Potential to eliminate long-lived PCs	Severe side effects including neuropathy might limit long term use and use in less severe cases
Autoreactive PCs	Immuno- Proteasome [167] e.g., KZR-616	Selective inhibition of inflammatory PC proteasome opposed to wide PC inhibition of first in-class proteasome inhibitors (Bortezomib)	Postulated selectivity	So far, rather experimental approach
PCs	CXCR4 [168] e.g., Mozobil (Plerixafor)	Dislocation of aberrant PCs from immune-privileged niches	High level of experience from therapy of hematologic malignancies	Rather theoretical approach for autoimmune disease Mechanistically, monotherapy unlikely to be sufficiently effective
(PCs)	IgG/FcRn [169] e.g., ARGX-113 (Efgartigimod)	Neutralization of global IgG including autoantibodies	No impairment of other immunoglobulins and albumin when compared to plasmapheresis Approach appears to be a safe alternative to apparative removal of immunoglobulins	No specificity towards aberrant/self-reactive immunoglobulins
(PCs)	Immuno- globulins (plasma- pheresis, immune- adsorption) [147]	Removal of pathogenic autoantibodies via exchange of blood plasma or selective removal of compounds by specific membranes	Well-established add on therapy to other therapeutic approaches High level of experience	Limited availability Invasive and time consuming procedure Severe side effects due to removal of albumins and other plasma proteins (hypoglobulinemia) by plasmapheresis lead to severe immunosuppression

Abbreviations: CD: cluster of differentiation; BM: bone marrow; TLS: tertiary lymphoid structure; BCR: B-cell receptor; CAD: cutaneous autoimmune disease; BLyS: soluble human B lymphocyte stimulator protein; BAFF: B-cell activating factor; BTK: Bruton’s tyrosine kinase; PI3Kδ: phosphatidylinositol 3-kinase δ; PCs: Plasma cells; SHIP1: rc homology 2 (SH2) domain containing inositol polyphosphate 5-phosphatase 1; ROCK2: Rho-associated protein coiled-coil containing kinase 2; IgG: immunoglobulin G.

**Table 2:** Exemplary clinical trials of B-cell- and PC-directed therapies in miscellaneous inflammatory skin diseases as of October 2020.

Drug Name	Target Structure	Condition	Phase	NCT Identifier
XmAb 5871 (Obexelimab) (humanized Fc engineered antibody)	CD19	SLE	II	NCT02725515 (completed)
Rituximab (chimeric antibody)	CD20 First in-class	Bullous pemphigoid	I/II III	NCT00286325 (completed) NCT00525616 (completed)
		Ocular cicatricial pemphigoid	I/II	NCT00584935 (completed)
		Mucous membrane pemphigoid	III	NCT03295383 (recruiting)
		Pemphigus vulgaris	II/III III II	NCT00213512 (completed) NCT01299857 (completed) NCT04400994 (recruiting; +IVIG)
		Dermatomyositis	II	NCT00106184 (completed)
Ocrelizumab (humanized antibody)	CD20	Systemic lupus erythematosus	III	NCT00539838 (terminated)
Obinutuzumab (humanized antibody)	CD20	Chronic GvHD	II	NCT02867384 (recruiting)
Veltuzumab (humanized antibody)	CD20	Pemphigus vulgaris	Case report only	
Ofatumumab (fully human antibody)	CD20	Pemphigus vulgaris	III III	NCT02613910 (terminated) NCT01920477 (terminated)
Belimumab (fully human neutralizing antibody)	BLyS (also named BAFF)	CLE	Pooled Analysis III	NCT01858792 (completed) BELI-SKIN: 2017-003051-35 (recruiting)
		Diffuse cutaneous systemic sclerosis	II	NCT01670565 (completed)
Atacicept (recombinant fusion protein)	BLyS/APRIL	SLE	II II/III	NCT02070978 (completed) NCT00624338 (completed)
A-623 (Blisibimod) (recombinant fusion protein)	BLyS	SLE	II III	NCT01162681 (completed) NCT01395745 (completed)
LY2127399 (Tabalumab) (human neutralizing antibody)	BLyS	SLE	III III	NCT02041091 (terminated) NCT01205438 (completed)
VAY736 (Ianalumab) (human antibody)	BLyS	Pemphigus vulgaris	II	NCT01930175 (completed)
Ibrutinib (oral irreversible inhibitor)	BTK First in-class	Chronic GvHD	III II	NCT02959944 (active, not recruiting) NCT04294641 (recruiting)
Acalabrutinib (oral irreversible inhibitor)	BTK	GvHD	II	NCT04198922 (recruiting)
PRN1008 (oral reversible inhibitor)	BTK	Pemphigus vulgaris	II III	NCT02704429 (completed) NCT03762265 (recruiting)
ABBV-105 (Elsubrutinib) (oral irreversible inhibitor)	BTK	SLE	II II	NCT04451772 (recruiting) NCT03978520 (recruiting)
Idelalisib (oral reversible inhibitor)	PI3Kδ First in-class	Allergic rhinitis	I	NCT00836914 (completed)
Parsaclisib (oral reversible inhibitor)	PI3Kδ	Pemphigus vulgaris	II	NCT03780166 (withdrawn)
UCB-5857 (Seletalisib) (oral reversible inhibitor)	PI3Kδ	Psoriasis vulgaris	I	NCT02303509 (completed)
AQX-1125 (Rosiptor) (oral reversible activator)	SHIP1	Atopic eczema	II	NCT02324972 (completed)
KD025 (Belumosudil) (oral inhibitor)	ROCK2	Diffuse cutaneous systemic sclerosis	II	NCT03919799 (recruiting)
		Psoriasis vulgaris	II II II	NCT02106195 (completed) NCT02317627 (completed) NCT02852967 (completed)
ARGX-113/ Efgartigimod (neutralizing human antibody)	IgG/FcRn	Pemphigus vulgaris and foliaceus	II	NCT03334058 (active, not recruiting)
SYNT001 (monoclonal IgG4 antibody)	IgG/FcRN	Pemphigus vulgaris and foliaceus	I/II	NCT03075904 (completed)
KZR-616 (oral irreversible inhibitor)	Immuno-proteasome	Dermatomyositis	II	NCT04033926 (recruiting)

Abbreviations: PC: Plasma cell; SLE: systemic lupus erythematosus; CLE: cutaneous lupus erythematosus; Fc: fragment crystallizable region; CD: cluster of differentiation; IVIG: intravenous immunoglobulins; GvHD: graft versus host disease; BLyS: soluble human B lymphocyte stimulator protein; BAFF: B-cell activating factor; APRIL: A proliferation-inducing ligand; BTK: Bruton’s tyrosine kinase; PI3Kδ: phosphatidylinositol 3-kinase δ; IgG: immunoglobulin G; FcRn: neonatal Fc receptor.
